# Differential accumulation of metals in the lacustrine and fluvial Alpine bullheads (*Cottus poecilopus*) and recovery of fish from metal contamination after a flash flood

**DOI:** 10.1007/s11356-024-32288-z

**Published:** 2024-02-10

**Authors:** Marián Janiga, Martin Janiga, Tatiana Pitoňáková

**Affiliations:** 1 Institute of High Mountain Biology, University of Žilina, Tatranská Javorina 7, 059 56, Žilina, Tatranská Javorina Slovakia; 2 Faculty of Humanities and Natural Scienes, University of Presov, Presov, Slovakia

**Keywords:** *Cottus poecilopus*, Alpine lakes and streams, Heavy metals, Biomagnification, bioaccumulation, the Tatra mountains

## Abstract

The spatiotemporal distribution and transport of mercury, zinc, molybdenum, rubidium, and strontium from alpine terrestrial ecosystems to alpine lake and mountain stream populations of *Cottus poecilopus* were investigated. Metals were measured for 66 wild fish collected from different lakes and Javorinka stream across. Mercury was measured in the pectoral fins, other elements in the skull. Bullheads contained more metals in the alpine lakes than in the mountain stream. In particular, mercury and zinc concentrations in lake bullheads were 6 and 2.5 times higher, respectively, than those of stream-dwelling fish. New data were generated on metal bioaccumulation in fish of understudied West Carpathian alpine lake environments. In July 2018, a major flood occurred in the area of the Javorinka. Already then, the mercury content in bullheads increased significantly. Bioaccumulation of mercury in fish occurred very quickly after the flood and was also significant in the following 2019. Then, the concentrations of mercury quickly decreased up to 70% in 2021–2022. Average concentrations of molybdenum and rubidium in bullheads in the stream rapidly declined in the year following the flood disturbance, but within less than 2 years, the metal levels stabilized at about the same level as in 2017 prior the flood. Strontium concentrations in fish dropped rapidly immediately after the flood, increased in the following years, and dropped again after 4 years, suggesting that many more factors are influencing strontium bioaccumulation in fish that are comparable in magnitude to the flood. The most serious warning seems to be the absence of biogenic zinc. The average concentration in the Alpine bullheads population in the stream has declined by 70% in less than 5 years and is steadily declining. An important result of this study is the demonstration that disturbance by a single factor (heavy rainfall and flooding) has a clear and timely effect on average metal concentrations in the fish population.

**Graphical abstract:**

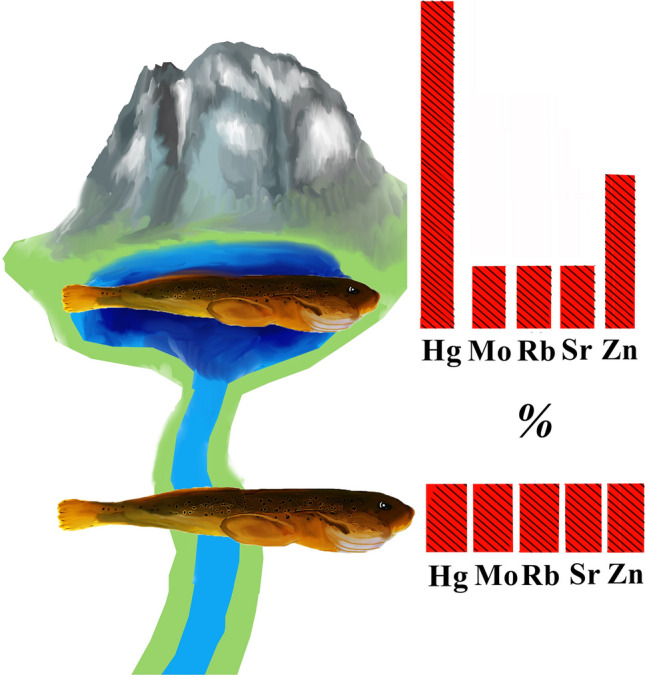

## Introduction

Many of the same fish species can survive in lakes but also in rivers, for example migratory fish that migrate from lakes and seas to spawn in rivers, but also resident fish that breed in situ. This includes the Alpine bullhead, *Cottus poecilopus*. The Alpine bullhead is a species of freshwater fish of Eurasian continent and in central and northern Europe is found in upland and cold-water streams and lakes. This species may be restricted to some lakes as a glacial relict of the late phases of the Vistula glaciation about 10,000 years ago (Kotusz et al. 2004). For example, in Lake Hancza (northeastern Poland), alpine bullheads use littoral habitats for spring spawning; in April and May, the density of bullheads in the littoral is highest. The rest of the year, *C. poecilopus* lives in Lake Hancza and Lake Luzin (northeastern Germany) mainly in profundal or sublittoral waters. Diurnal vertical movements of this species have been observed. During the day, most of the fish were at depths of 28 m and deeper, while at dusk and at night, the fish moved higher, to depths of 16 m (Kotusz et al. 2004). All these lakes were characterized by the dominance of stony bottoms in the littoral and sandy and muddy bottoms in the subliteral and profundal zones.

**Fig. 1:**
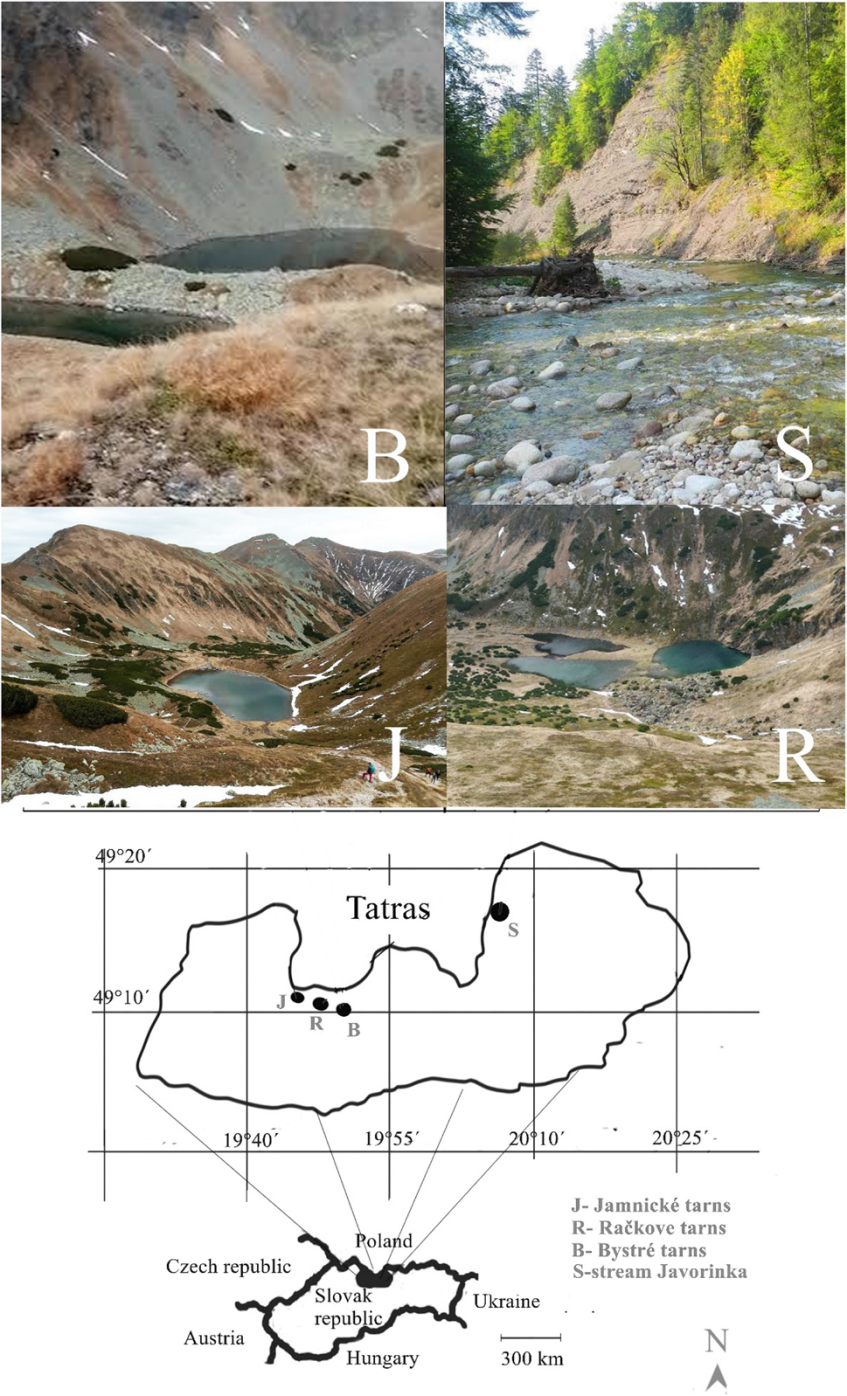
Sample locations of the lake and stream alpine bullheads in the Tatra mountains

Ecological requirements of lacustrine and fluvial bullheads differ, lacustrine occur in the marginal or deep parts of lakes (Huet 1959), fluvial in streams in relatively unpolluted water with high oxygen saturation (Janiga Jr 2018). In the sister species *Cottus gobio*, fluvial populations produce a small number of large eggs from which the benthic larvae hatch (Smyly 1957; Fox 1978) while lacustrine animals from the Hallstättersee Lake in Austria produce pelagic larvae (Wanzenböck et al. 2000). Fluvial populations mostly produce large eggs in the upper reaches of rivers, from which large well-developed larvae hatch. Immediately, after hatching, they assume a benthic life, in the upper and middle parts of the rivers. Food availability for the fry is low, but predation pressure is also low (Greenberg 1964; Goto 1990). Fluvial bullheads are a shallow-water sedentary species that hides under rocks (Welton et al. 1983; Janiga Jr 2018). From non-river water formations, alpine bullhead is known to occur in coastal and tributary brackish waters of the Black sea (Slastenenko 1955) or in mountain lakes (Bitušík et al. 2006). The lakes of the Tatra Mountains were, with few exceptions, originally fishless; historical brown trout (*Salmo trutta*) and alpine bullhead stocking in Račkove, Jamnické and Bystre lakes (Fig. 1) could have been started at the end of the eighteenth century (Bohuš 1977, 2005; Bitušík and Hamerlík 2022). The establishment of the Tatra National Park in 1948 led to the limitation of stocking; the brown trout probably went extinct and alpine bullhead expanded (Slobodníková et al. 2023).

The different life strategies of lake and river bullhead populations are undoubtedly influenced by different levels of water pollution and the accumulation of different contaminants in their diet. These contaminants include metals such as Hg, Zn, Rb, Sr, and Mo which especially at higher levels seriously affect the life of the species in the mountains (Perinajová et al. 2020; Janiga et al. 2021; Janiga Jr and Janiga 2023). Because of its ability to methylate, one of the most serious contaminants is mercury. In high mountain alpine lakes, alpine rivers, and aquatic environments, two types of mercury in fish have been detected: inorganic mercury and methylmercury (MeHg or CH_3_Hg^+^ ). Methylmercury is the most toxic form of mercury for fish and causes several problems in fish growth including liver damage and overall poor body condition (Simoneau et al. 2005; Drevnick et al. 2008). Reduced feeding, reduced ability to catch prey, or reduced ability to avoid predators and other changes in fish behavior due to mercury poisoning have also been reported (Weis 2009). Methylmercury is formed from inorganic compounds by the action of methanogenic bacteria in anaerobic environments, e.g., in sediments of fresh and saline waters. Because it is soluble in water and in streams, it remains in the aquatic environment from which it readily passes into fish and fish meat (Kafka and Puncocharova 2002). Carnivorous and piscivorous fish exhibit higher Hg levels than herbivorous and omnivorous, and larger fish of the same species generally contain more Hg than smaller ones (Malm et al. 1997). Predatory fish usually have a higher Hg content in comparison with fish that feed on phytobenthos and plants (Beltran-Pedreros et al. 2011). Hg bioaccumulation in fish predators also can be affected because of differences in available diet (MacCrimmon et al. 1983). Mercury currently is included with rubidium as metal that consistently biomagnifies in diverse food webs (Campbell et al. 2005). Rubidium is a cation K^+^ analog and can compete with K^+^ for enzymatic sites in fish and other organisms (Peters et al. 1999). Biomagnification of rubidium is significant within a food-web context, but not necessarily within species. Rubidium does not biomagnify as rapidly as ubiquitous methylmercury, which forms stable protein complexes. However, the consistent biomagnification of rubidium in food webs indicates that this alkali metal should be considered along with mercury and other organic and inorganic contaminants when studying biomagnifying compounds in food webs (Campbell et al. 2005).

### Zinc, strontium, and molybdenum in fish

The main role of zinc is based on its function as a part of metalloenzymes for controlling the activity of specific zinc-dependent enzymes. High intakes of zinc in relation to cuprum can cause a cuprum deficiency (Maret and Sandstead 2006; Mielcarek et al. 2020). Zinc is also known to biomagnify in certain ecosystems (Quinn et al. 2003). Anthropogenic activities, such as agriculture, urbanization, and mining, have resulted in elevated levels of zinc in natural waters. Strontium is mainly present in the dissolved phase of aquatic systems, and its concentrations are generally higher in marine than freshwater ecosystems. It has been found to accumulate in the bony tissues of fish, especially in soft waters, and is readily incorporated into fish otoliths (“earstones” made of calcium carbonate) (Chowdhury and Blust 2011). Molybdenum is an important element in the production of alloy steels and chemicals. Most natural waters exhibit conditions where the stable species of molybdenum is generally the molybdate ion. Molybdate is the main form of estimation of the toxicity of molybdenum in an environment (Jones et al. 1994).

The Alpine bullhead appears to be a good indicator of the overall condition of the habitat, and especially newly hatched small bullheads are considered bioindicative of water purity in mountain creeks, streams, or rivers (Janiga Jr and Janiga 2023). In *Cottus* fish, a wide variety of effects due to inorganic and mainly organic effluents were detected, in the span from physiological changes to disorders in reproduction (Bucher et al. 1992; Baruš and Oliva 1995; Janiga Jr and Janiga 2023). Concerning the heavy metals and other pollutants in fish of mountain waters, their manifestation radically differs between lakes and rivers (Dočkalová et al. 2015; Shao et al. 2016; Janiga et al. 2021; Riaz et al. 2021). In addition to the annual spring increases in water flows in mountain rivers, flashfloods have a significant impact on stream chemistry. Future scenarios for the West Carpathians assume an overall lower total precipitation but an increase in total precipitation in episodic situations in summer (Pecho et al. 2010; Pecho et al. 2009). The most extensive flood in the Tatras was recorded in 1958, and a similar situation was repeated in the stream Javorinka in July 2018 (for detailed information on this flood event, see Hrivnáková et al. 2020; Janiga et al. 2021). New evidence exists for local cyclonic circulation types of rainfall (Niedźwiedź et al. 2015) to which mountain species of fish are not well adapted. Change in stream morphology reduces the number alpine bullheads; the abundance of their diet may decrease to 95% (Kubín et al. 2018, 2019). Floods mobilize nutrients and other chemicals in mountain lakes and significantly affect biotic environment of mountain stream ecosystems (Wantzen et al. 2008; Hrivnáková et al. 2020). In many species of fish, the negative ecological footprint due to flash floods can still be observed several years following the event. The main objective of this study was to determine what is the bioaccumulation potential of Hg, Rb, Sr, Mo, and Zn in bullheads after a large flood, in fish living in the wild environment of a high mountain stream. The second aim was to determine how the concentrations of these metals in bullheads differ between alpine lake and mountain stream populations.

## Material and methods

### Field collection

Alpine bullheads have been collected from the following alpine lakes in the Tatra mountains: Veľké Bystré (49°10.96368′ 19°50.57322′,1879 m.a.s.l.), Dlhé Bystré (49°10.90042′ 19°50.56888′, 1879 m.a.s.l.), Nižné Jamnické (49°12.15184′ 19°46.19049′, 1732 m.a.s.l), and Vyšné Račkove (49°11.99569′ 19°48.30753′, 1697 m.a.s.l.). For a more detailed description of the hydrochemistry and hydrobiology of these lakes, see, for example, studies by Krno et al. (2006) or Bitušík et al. (2006). Due to the closed presence of metamorphic rocks, the lakes have relatively high concentrations of base cations, resulting in higher alkalinity than many other Tatra lakes (Krno et al. 2006). Sampling was carried out in October (14 dead animals) and early November 2022 (one individual). They were collected by hand. Sightings of live alpine bullheads have also been recorded. Dead bullheads from Javorinka stream were collected from January to December over a 6-year period from 2017 to 2022. Although the Javorinka stream is relatively distant from the mentioned lakes, the alkalinity of the streams coming from the lakes at the same altitude (860–950 m a.s.l.) and in the same season (August) is approximately at the same level: pH—Javorinka stream, 7.7; Račkov stream, 7.2; Bystrý stream, 7.2 (Gura et al. 2013; Hrivnáková et al. 2020). Size in adult bullheads affects bioaccumulation of chemical elements only minimally, but significantly higher values were found in fry (Janiga Jr and Janiga 2023). Specimens smaller than 40 mm were excluded. From the stream, a total of 137 presumably adult individuals were used for our analysis. Geomorphology of Tatra valleys, lakes, and rivers is a result of last Holocene temperature variations. To compare the levels of accumulated chemical elements in fish between the lakes and Javorinka stream, 51 October samples from the stream were used. All dead alpine bullheads were taken in plastic bags; the geographical coordinates and altitude of their occurrence were recorded.

### Laboratory analyses

Alpine bullhead samples were weighed using a Kern 770 analytical spring balance (Kern, Germany) and measured from the tip of the head to the end of the caudal fin (Baruš and Oliva 1995). After the measurements, bullheads were placed in freezer boxes; they were stored in a freezer at − 20 °C. For fast and accurate measurement of concentrations of chemical elements in the skull bones, a fluorescence ED-XRF spectrometer DELTA (BasRudice s.r.o., CZECH) was used. Standardization of the instrument with Standard 316 was performed once every 24 h. In the spectrometric device, each element is identified according to the wavelength or energy of radiation emission. The wavelength is inversely proportional to the energy and is characteristic of each element. Measurement accuracy and standard: bone ash (NIST 1400, Maryland, USA). The samples were handled in the same way, the scanning window of the XRF spectrometer was attached to the parietal part of the fish’s head. The thickness of the measured material was always greater than 5 mm. Byrnes and Bush (2016) report a critical value for bone samples of 1.9 mm. Samples were analyzed by multiple-beam measurement; every measurement consisted of three beams for 80 s, repeated three times and averaged. For methodology, see details in Janiga Jr and Janiga (2023). In spectrometry, the quality assurance depends on determination of the lowest limit of detection (LOD). We used factory calibration, based on the Compton normalization (CN). The CN algorithm is designed for achieving the exact LOD (Innov-X Systems 2010). The LODs were determined for each measurement and for elements S, Cl, K, Ca, Zn, Rb, Sr, and Mo.

For mercury analysis, we cut the right or left pectoral fin, approximately 40 mm from the proximal end. The fins were dried at 40 °C for 12 h. Mercury concentration in fins was measured on a DMA-80 Direct Mercury Analyzer (Milestone, Italy). The accuracy of the measurement was verified by analysis of standard reference material including NCS ZC 71001 beef liver (CHNACIS, China). The detection limit of our instrument is 0.003 ng of Hg. The limit of detection (LOD) and the limit of quantification (LOQ) were established by the 10 independent analyses of the blank (empty nickel boat). The following equations were used to calculate LOD and LOQ: LOD = 3 * s and LOQ = 10 * *s*, where *s* = average standard deviation, calculated from 10 independent determinations of the blank. LOD and LOQ were determined as 0.0037μg/g and 0.0012 μg/g, respectively. Our method represented a high average % recovery (> 95%) in the standard reference material matrix and a relative standard deviation (RSD) less than or equal to 5%.

### Statistics

Differences in mercury levels among groups were compared by nonparametric Mann-Whitney and Kruskal-Wallis tests, with a significance level of *p* < 0.05. Principal component analysis (PCA-software PAST, version 4.12) was used to compare the scores of multivariate vectors (component weights) of original data in relation to element concentrations in bullheads from different habitats. The scores were compared by one-way ANOVA.

## Results

Levels of all metals in the alpine bullheads were higher in the lakes than in the mountain stream. Statistically significant differences were especially in mercury and zinc levels. Fish in lakes contained up to six times more mercury than fish from mountain stream. Zinc content was 2.5 times higher in bullheads from lakes than in fish from Javorinka stream. Levels of rubidium, molybdenum, and strontium did not differ between fish in lakes and streams, suggesting their approximately equal sedimentation patterns in rivers and lakes (Table 1).

**Table 1:** Mean concentrations of Hg in pectoral fins and of Mo, Rb, Sr, and Zn in skulls of alpine bullheads from the Tatra mountains, the West Carpathians. The samples of adult fish originate from October. Localities are shown in Fig. 1. The medians were compared by Mann–Whitney *U* test (*M*-*W U* test)

Element (mg/kg)	Habitat	Mean (± SE)	*n*	*M*-*W U* test (*z*)	*P*
Hg	Lake	0.047 (0.004)	15	− 4.9	0.0000 ***
	Stream	0.008 (0.002)	51		
Mo	Lake	1.8 (0.13)	15	− 1.5	0.12 Ns
	Stream	1.52 (0.07)	51		
Rb	Lake	4.09 (0.51)	15	0.7	0.46 Ns
	stream	3.82 (0.28)	51		
Sr	Lake	57.2 (5.3)	15	− 1.8	0.07 Ns
	Stream	41.06 (2.9)	51		
Zn	Lake	49.2 (6.08	15	− 3.9	0.0000 ***
	Stream	20.6 (3.3)	51		

**Fig. 2:**
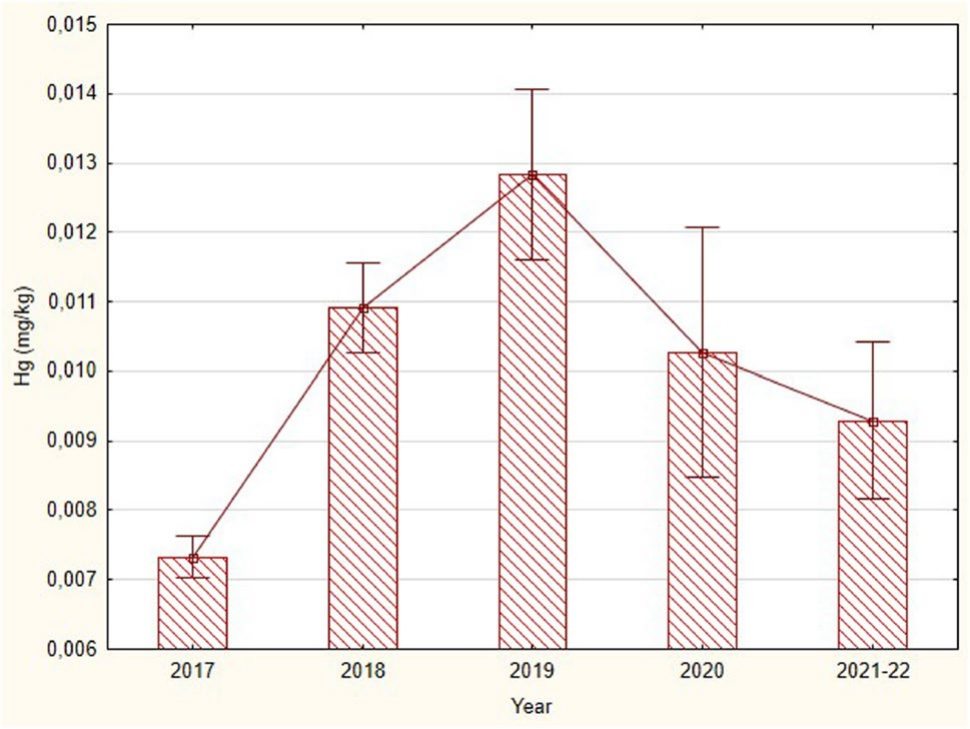
Means (± SE) of total mercury concentrations (wet weight) in pectoral fins of alpine bullheads (longer than 40 mm) collected from the stream Javorinka in the years 2017–2022. The significant differences were found between the years 2017 and 2018 and between 2017 and 2019 (Kruskal-Wallis test: *H* (4.133) = 24.3, *p* = 0.0001). The large flood occurred in July 2018. The majority of bullheads from 2018 were collected after flood. The amount of Hg increased rapidly 1 year after the flood (2019); in 2021–2022, the concentration of mercury in this small-bodied fish continually decreased by up to approximately 70 %

In July 2018, a major flood occurred in the area of the Javorinka stream in the Javorová valley. Already this year, the mercury content in fish increased significantly compared to 2017 (Fig. 2). Most of the 2018 samples were taken after the flood. This means that the bioaccumulation of mercury in fish occurred very quickly after the flood and was also significant in the following year, 2019. It was not until 2021–2022 that mercury levels stabilized at about the same level as in 2017, indicating that the recovery time in fish was more than 2 years after the flood.

**Fig. 3:**
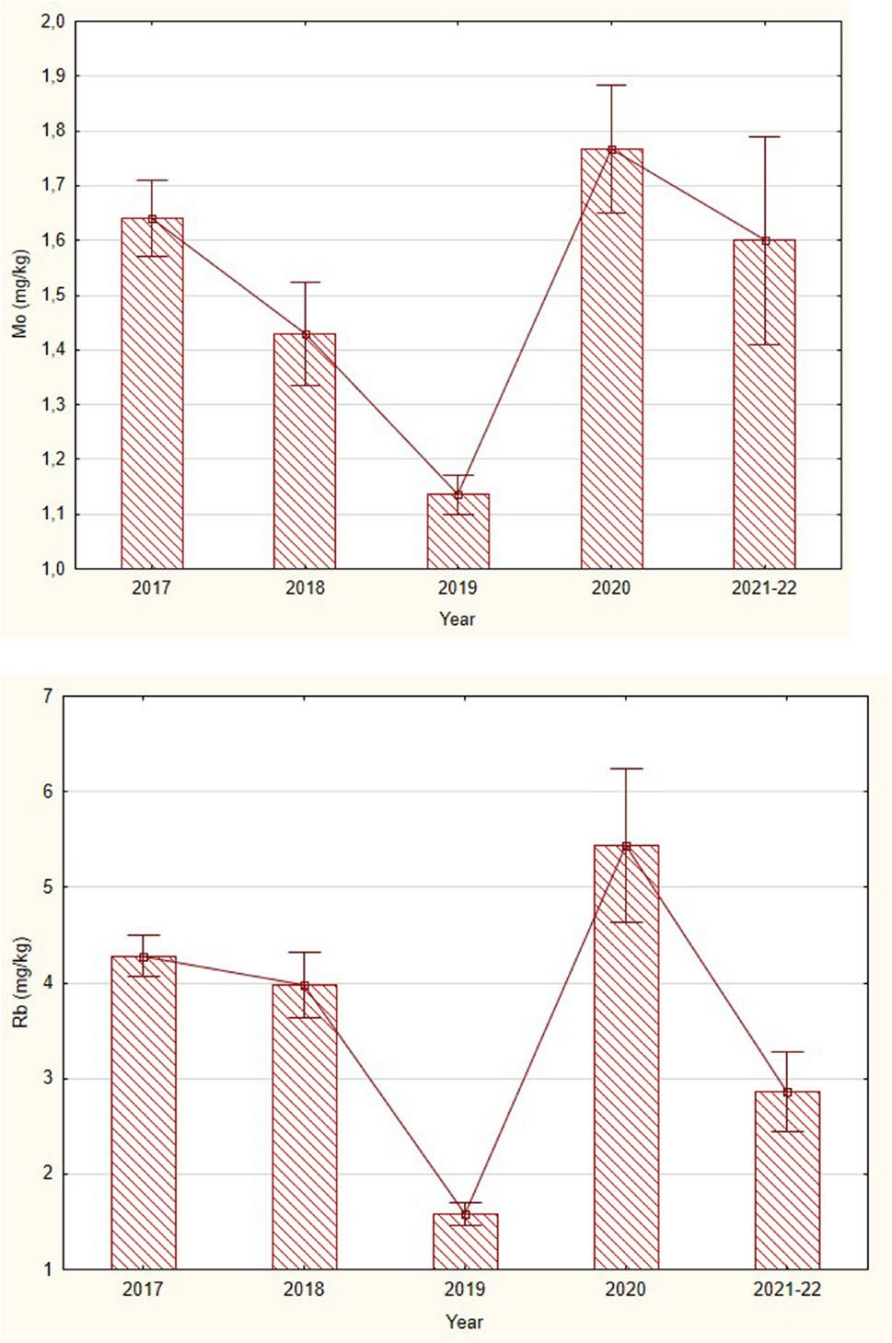
Mean (± SE) concentrations (wet weight) of molybdenum (top) and rubidium (bottom) in the skulls with skin of alpine bullheads (longer than 40 mm) collected from the stream Javorinka in the years 2017–2022. Mo—KW-H (4120)= 41.04, *p* = 0.0000, Rb—KW-H (4,133) = 65.5, *p* = 0.0000

Molybdenum and rubidium concentrations behaved in approximately the same pattern during the flood. The 2018 summer flood has not yet affected the concentrations of metals in bullheads because the levels of these metals did not differ between 2017 and 2018 (Fig. 3). A sharp decline in both elements in bullheads bodies occurred the following year, 2019. Between 2020 and 2022, levels of both rubidium and molybdenum increased significantly with a decline towards 2021–2022. Recovery time in fish bodies for these metals can be estimated to be 1 year after the large flood.

**Fig. 4:**
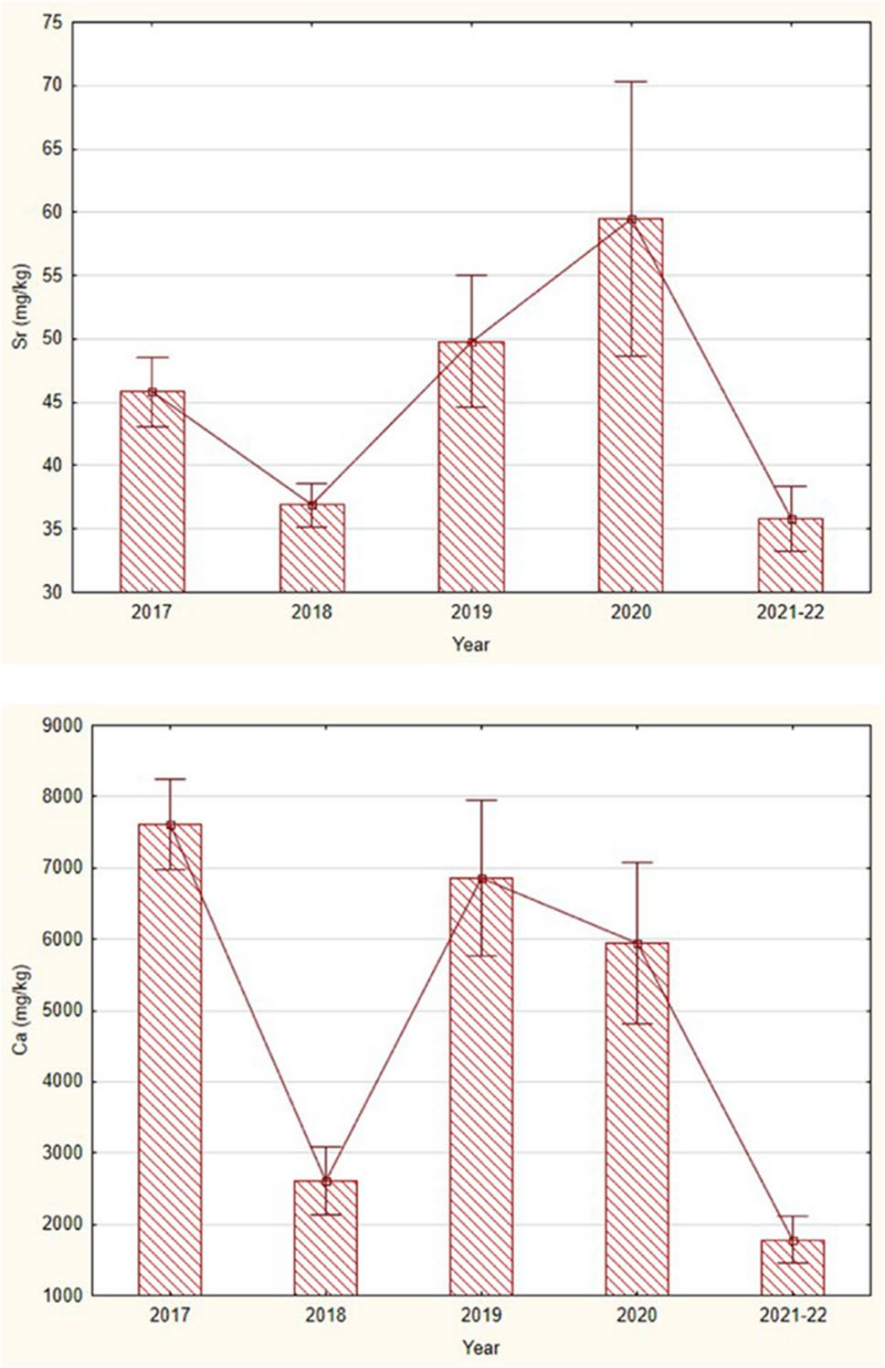
Mean (± SE) concentrations (wet weight) of strontium (top) and calcium (bottom), in the skulls with skin of alpine bullheads (longer than 40 mm) collected from the stream Javorinka in the years 2017–2022. Sr—KW-H (4133) = 9.81, *p* = 0.04, Ca—KW-H (4,133) = 34.3, *p* = 0.0000

Strontium concentrations in fish were low in the 2018 flood year. The values were also very low in the years 2021–2022 (Fig. 4). From the above, it can be assumed that the accumulation of strontium in fish is very little affected by floods, or there are other factors that affect the accumulation of strontium in this fish species as well as the flood. Strontium is probably more sensitive to seasonal and annual environmental changes; the variation ran parallel with calcium (Fig. 4). In water, an increase of the calcium concentrations is generally accompanied by an increase also in the strontium concentrations. Differences between these two elements in fish bodies seem to depend more on seasons than on habitat type (Table 1).

**Fig. 5:**
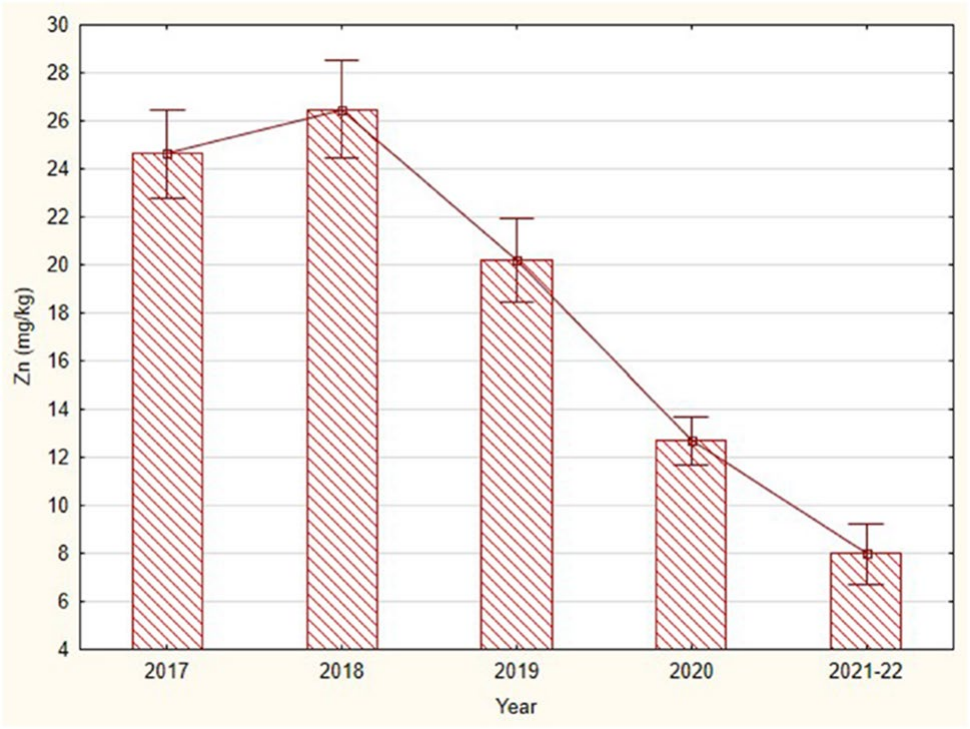
Mean (± SE) concentrations (wet weight) of zinc in the skulls with skin of the Alpine bullheads (longer than 40 mm) collected from the stream Javorinka in the years 2017–2022 (KW-H (4133) = 48.53, *p* = 0.0000)

Probably the most serious impact of flooding is observed for zinc. From steady-state levels in 2017 and 2018, zinc in fish declines rapidly (Fig. 5). This means that the large flood in July 2018 caused a long-term absence of zinc in bullheads in streams, and as of 2022, a recovery time could not yet be determined. An important result of this study is the demonstration that disturbance by a single factor (heavy rainfall and flooding) has a clear and timely effect on metal concentrations in fish populations, even for short-lived bullheads that are only slowly recovering the levels of some biogenic elements (Zn) in their bodies.

**Fig. 6:**
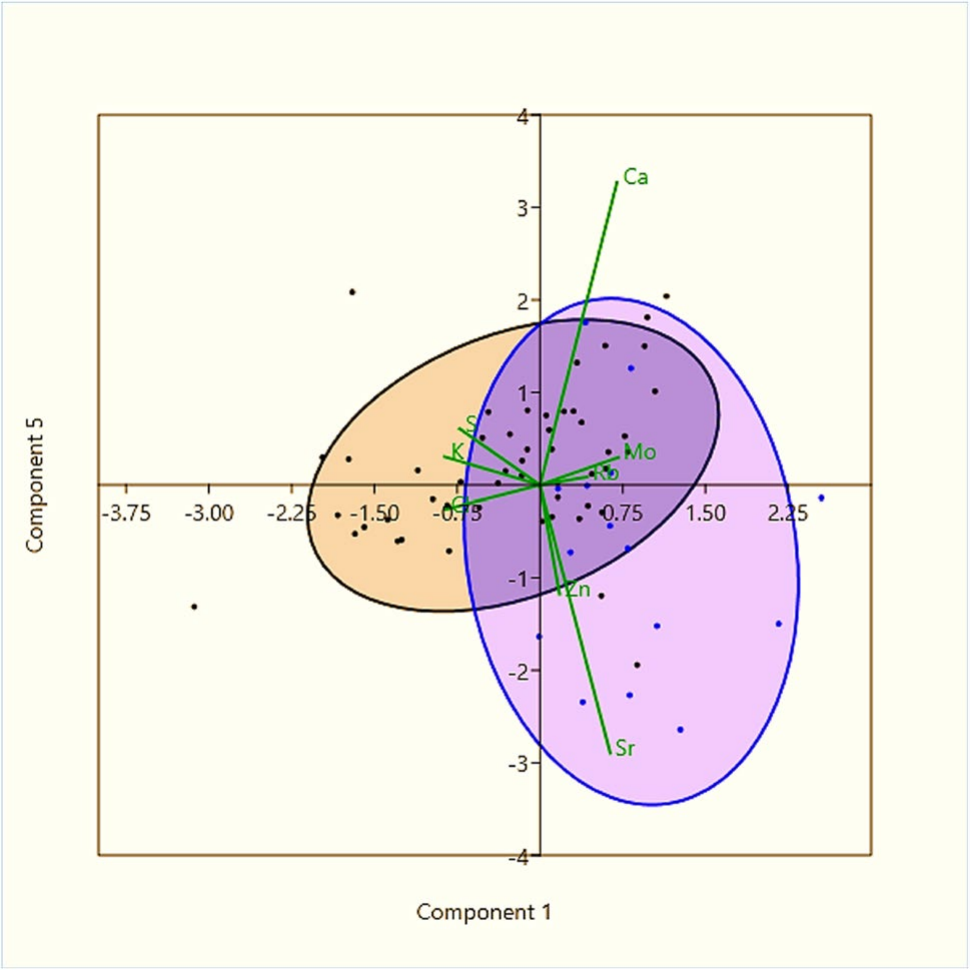
Differences in mutual accumulation of some elements in lacustrine (blue, right ellipse) and stream (brown—left ellipse) bullheads in the Tatra mountains. The ellipses express 95% confidence limits. Stream bullheads had proportionally more sulfur, chlorine, and potassium (x-axis left) and less calcium, rubidium strontium, and molybdenum in the skulls. In lacustrine individuals, it was *vice versa* (*x*-axis right). Component 1: One-way ANOVA *F*(1, 64) = 16.5, *p* = 0.0001, and the two groups differed significantly. The bullheads also significantly differed in another phenomenon, namely, in the way of strontium and calcium accumulation. In lacustrine fish, the ratio of strontium to calcium was higher (*y*-axis down) than in fish living in the stream Javorinka (*y*-axis up). Component 5: One-way ANOVA *F* (1, 64) = 11.7, *p* = 0.002

The different phenomena of mutual accumulation of elements in fish are shown in Table 2 and Fig. 6. The first most significant phenomenon (PC1, Table 2) describes the difference between the accumulation of elements in stream- and lake-dwelling fish. Macrominerals sulfur, potassium, and chlorine rapidly leach from lakes into streams, while calcium, rubidium, strontium, and molybdenum remain more in lakes and are absorbed by bullheads in that ratio, more macrominerals in the stream and more metals in the lake (*x*-axis in Fig. 6). The second, third, and fourth phenomena are not related to differences in habitat, but to the way in which fish accumulate metals after a flood and how they dispose of them. The second component (PC2) describes similar annual trends in concentrations of rubidium and molybdenum in bullheads (Fig. 3); the third, independent variability in zinc accumulation (Fig. 5); and the fourth, mutual accumulation of calcium and strontium (Fig. 4). These phenomena are likely to occur equally in both streams and lakes (Table 1). The last fifth phenomenon (PC5) reflects known facts from the mutual accumulation of strontium and calcium in bones in calcium-deficient environments. An increasing calcium concentration in water tends to lower strontium content in bullhead bone in streams, while strontium/calcium ratios in fish in lakes are higher (y-axis in Fig. 6).

**Table 2:** The first five components and their weights of the principal component analysis (PCA) which identify major trends in the concentrations of chemical elements in the Alpine bullheads. Macrominerals S, Cl, K, and Ca were included in the analysis. The numbers in bold and their signs indicate for each component the trend in the chemical composition of the major elements

Element	PC 1	PC 2	PC 3	PC 4	PC 5
S	**− 0.382**	0.235	0.317	0.303	0.132
Cl	**− 0.450**	0.290	− 0.174	0.329	− 0.057
K	**− 0.454**	0.282	− 0.138	0.157	0.066
Ca	**0.366**	− 0.070	− 0.042	**0.572**	**0.712**
Zn	0.093	− 0.038	**0.857**	0.247	− 0.255
Rb	**0.224**	**0.689**	0.086	− 0.184	0.018
Sr	**0.334**	0.064	− 0.324	**0.578**	**− 0.630**
Mo	**0.372**	**0.542**	0.011	− 0.132	0.064
Variance	38%	19%	14%	12%	6.5%

## Discussion

The oligotrophic mountain lakes are permanently exposed to flooding, the intensity and season of which is changing with global warming (Niedźwiedź et al. 2015; Hrivnáková et al. 2020). This Alpine bullhead currently lives in several alpine lakes as well as in mountain streams of the Tatra mountains, the West Carpathians. The aim was to provide further insights into how the system “wet and dry deposition—atmospheric pollution—mountain lakes—intense precipitation—floods—mountain streams—transfer of deposited chemical elements from lakes to streams” affects the chemical composition of food webs in mountain watercourses and also what the recovery time in stream ecosystems after large floods is. Trace elements occur in the atmosphere in the form of solid particles or are adsorbed onto them, and these atmospheric aerosols can travel long distances before being redeposited into the alpine lakes (Patterson and Settle 1987).

### Mercury

Mercury is considered a major environmental contaminant worldwide due to its complex biogeochemistry in nature, high atmospheric deposition, a strong tendency of biomagnification, and high potential toxicity (Xu et al. 2019; Santos et al. 2021). More than 80% of mercury in terrestrial ecosystems comes from atmospheric inputs via wet and dry deposition (Jiskra et al. 2015; Zhang et al. 2015). Atmospheric deposition of mercury increases with altitude (Blais et al. 2006; Wang et al. 2017), with its concentrations in the alpine habitats approximately twice as high as in the lowlands (Ballabio et al. 2021). The form in which it is deposited is Hg^2+^; in water, it is efficiently converted to methylmercury (MeHg), which in turn is efficiently taken up by aquatic organisms in the first stages of the food chain (Zillioux 2015). Spring flooding of snowmelt in elevated alpine lake areas is reported as important mercury source, releasing to the lake water, and sediments could be another reason of mercury important source (Loseto et al. 2004; Chételat et al. 2015). The concentration of mercury ions is usually different in different water layers, especially at the sediment-water interface (Zoumis et al. 2001). Transport velocity for trace metals associated with settling particles or movement with water is reduced by many orders of magnitude across this interface (Santschi et al. 1990). Metals are usually trace in water, but are often many times higher in sediments than in water (Begum et al. 2009), sometimes up to several orders of magnitude (Camarero et al. 2009). Overall, the Tatra lakes are among the more polluted high mountain lakes in Europe (Dočkalová et al. 2015). Besides the availability of mercury in water or sediments (i.e., the production of methylmercury), factors influencing mercury concentrations in fish include the bioaccumulation rate, e.g., uptake, excretion, and growth (Wang 2012) and the biomagnification of MeHg through the food web (Downs et al. 1998). Much remains unknown about the feeding strategies of alpine bullheads in the Tatra small lakes. The food biomass of adult fish living in the Tatra streams consists mainly of mayflies (family Baetidae) and caddisflies (Hydropsychidae), but the fish often catch larvae of true flies (midges—Chironomidae and blackflies—Simulidae—Števove et al. 2019). Pastorino et al. (2020a, 2020b, 2020c) found that in Dimon Lake in the Carnic, Alps bullheads (*Cottus gobio*) feed exclusively on Chironomidae larvae all year round, and so the authors assume that the amount of ingested metals from diet originates from midges. In stream systems, mercury accumulates in Chironomidae in a similar pattern to zinc (Pastorino et al. 2019a). In alpine lakes, zinc concentrations in midges are higher than in lake sediments or bullhead tissues (Pastorino et al. 2020c), so it can be assumed with high probability that Chironomidae are also the main source of mercury in fish from the Tatra lakes. Chironomids are excellent bioindicators to investigate the trophic state of lakes over the course of hundreds of years (Slobodníková et al. 2023). In these lakes, the genera *Paratanytarsus*, *Heterotrissocladius*, *Corynoneura*, *Zavrelimyia* are the most abundant (Bitušík et al. 2006; Krno et al. 2006). Regarding the feeding functional groups, grazers/scrapers and collectors dominate the lakes for more than 200 years and do not show marked changes over time (Slobodníková et al. 2023). There is a relatively high abundance of algae in these lakes, which (Bitušík and Hamerlík 2022) attributes to the low abundance of macrozoobenthos, which feed on algae. Even 200 years after the fish were released into the lakes, besides midges, several species of aquatic invertebrates undoubtedly lived in homeostasis with bullheads (Krno 1991). Some species are certainly still being fed on by adult fish today. They are mainly algophages and detritophages: *Nemoura monticola*, *Acrophylax vernalis* (rare and protected alpine species), *Chaetopteryx fusca*, *Ameletus inopinatus*, or *Hydroporus incognitus* (Krno 1991; Krno et al. 2006). Littoral macroinvertebrates and terrestrial insects, deposited by currents over the surface of lakes during the ice-free period, and benthic macrozoobenthos are probably a substantial food source for bullheads. The fish in these lakes contained up to six times more mercury in the pectoral fins than alpine bullheads in the Javorinka stream.

The higher mercury concentrations are probably the result of the synergistic effect of several factors. *First,* lifestyles of lake and stream bullheads differ. As mentioned in the “Introduction,” lake populations occur in the marginal or deep parts of lakes (Huet 1959). In summer, bullheads use littoral habitats for spawning and feeding. The rest of the year, they probably live in profundal or sublittoral waters with possible diurnal vertical movements. In stream specimens, the liver lipid contents start to decrease in summer, whereas in lake specimens, it increases, with a significant rise between July and August, at which time there is also an increase in water temperature and abundance of larvae of midges (Pastorino et al. 2019b). Fish inhabiting mountain lakes have to survive extreme environmental conditions, with low water temperature, long ice-covered periods, low ionic conditions, and low productivity in the aquatic habitat. The smallest depth of the lake, in which they are surviving the winter, is 8 m. They probably more feed on small benthic invertebrates. Although in oligotrophic systems pelagic pathways tend to be more efficient for mercury accumulation than benthic habitats (Power et al. 2002; Karimi et al. 2016), habitat-specific fish food is an important factor in the food web for mercury in fish living in alpine lakes. Numerous studies have found that food segregation—benthic versus pelagic—has significant effects on fish mercury concentrations. Fish with benthic diets tend to have higher mercury concentrations (France 1995; ; Andreji et al. 2006; Noël et al. 2013; Andreji and Dvorak 2018; Chiapella et al. 2021). Alpine bullheads in Tatra lakes are probably more profundal benthivores, while in Javorinka stream, they are littoral benthivores. Littoral benthivores contain much less mercury in their bodies than fish that feed on benthos from the profundal (Piro et al. 2022). *Second,* the low-temperature climate in high mountain areas can severely limit the elimination of methylmercury in fish (Zhang et al. 2014). Fish from mountain lakes generally grow very slowly, probably due to freezing temperatures or poor food quality and availability in the oligotrophic high mountain environment. The long life span and slow growth rate may be an important factor contributing to increasing concentrations of metals and other pollutants in fish (Dočkalová et al. 2015). *Third,* numerous evidence suggest foraging habitat and lake morphometry can have an outsized influence on mercury exposure, although the relative importance of different habitats can vary across the landscape (Eagles-Smith et al. 2016). *Fourth,* the accumulation of mercury in fish bodies is significantly affected by water chemistry. Lange et al. (1993) reported that uptake of MeHg in largemouth bass in Florida lakes was shown negatively correlated with alkalinity, calcium, chlorophyll ɑ, conductance, magnesium, pH, total hardness, total nitrogen, and total phosphorus. Sulfur also significantly inhibits methylation (Gabriel et al. 2014). In contrast, enhanced and stimulated mercury methylation by sulfate-reducing bacteria at low pH is considered the major reason responsible for the elevated fish mercury levels in acidified lakes. Methylation rates are faster in warmer temperature (Grieb et al. 1990; Dijkstra et al. 2013). However, the chemistry of alpine lakes is most influenced by high rainfall and subsequent flooding.

Following the extreme rainfall in July 2018, the pH level in alpine lake Kolové pleso increased, and the lake water became less acidic. The levels of some elements (S, K, Rb, Mo, Cd) decreased sharply after the flood disturbance. The lake was literally “cleaned,” with elements apparently moving into the streams and estuaries below the lake. The concentrations of these elements and mercury in the stream increased after the flood (Hrivnáková 2019; Hrivnáková et al. 2020; Janiga et al. 2021). Stream bullheads probably absorbed metals into their bodies through the trophic web. The bioaccumulation rate of mercury in fish after the flood was more than twice as fast as the elimination rate. The recovery time for alpine bullhead from mercury intoxication is approximately 2 years. Many authors report different recovery times for different fish species (Trudel and Rasmussen 1997; Jillian et al. 2007; Madenjian et al. 2021). The physiology of individuals, the shorter age of fish in streams than in alpine lakes, thermal preferences, and linkage to benthic habitats may play an important role and likely contribute to the recovery time from mercury intoxication in streams (Blanchfield et al. 2022). Seventy to 100% of mercury present in the fish body is of methylated form. Mercury has a high affinity to proteins; therefore, more than 90% of total mercury accumulates in fish muscle and blood (Garai et al. 2021). Although the amount of mercury measured in our study is in the pectoral fins and not in the muscles, we believe that the Tatra National Park ecosystems still provide a very clean environment for the life of this fish species. Levels not only in the stream but also in the lakes are significantly lower than, for example, those measured by Andreji and Dvorak (2018) in the muscles of alpine bullheads living in urbanized environments (0.05 to 0.12 mg/kg, Nitra River). In fish, the smallest amount of accumulated Hg is usually found in the gills, whereas the maximum concentrations are located in the muscle. Bones contain about one-third less mercury than muscles (Suhendrayatna et al. 2019).

### Zinc

The zinc cycle in the high mountains is very similar to the mercury cycle. It is one of the trace elements that can be transported long distances through the atmosphere and deposited in remote alpine regions (Kyllönen et al. 2009; Dong et al. 2015). It accumulates in sediments (Espinoza-Quiñones et al. 2011), in alpine lakes (Dočkalová et al. 2015), but mainly in phyto- and zooplankton (Pawlowski and Harasimiuk 1990; Parsons et al. 2001). The highest amounts of zinc are mainly in the easily and moderately reducible sediments; metals in these fractions are relatively available to be rereleased into the water environment. The metal is one of the most accumulated chemical elements in omnivorous carpids, especially in common carp, a species that apparently accumulates this element to a greater extent than other fish (Lowe et al. 1985; Andreji et al. 2006; Andreji and Stráňai 2007). Small fish species may be also sensitive to high levels of zinc in waters (Koca et al. 2005; Danabas and Ural 2012; Pastorino et al. 2020a). Fish can absorb zinc through the epithelial or mucosal surface of their skin (Xu et al. 2014), gills, and gastrointestinal tract, and the uptake of zinc from freshwater occurs mainly through gills by calcium-mediated pathway, and intestinal zinc uptake takes place mainly by carrier-mediated pathway (Hogstrand et al. 1994, 1995; Bury et al. 2003; Pyle et al. 2005; Dočkalová et al. 2015; McRae et al. 2016). In addition to the skin and gills, large amounts of zinc accumulate in the liver, bones, and kidneys, less in the gonads and intestine, and least in the muscles (Satoh et al. 1987; Stranai and Andreji 2005; Staniskiene et al. 2006; Espinoza-Quiñones et al. 2011; Rosseland et al. 2017).

In alpine lakes, bullheads are more likely to survive than salmonids; salmonids are probably some of the most sensitive fish to high levels of zinc in the aquatic environment (Sinley et al. 1974; Lahnsteiner et al. 2004; Andreji and Dvorak 2018). Similar to mercury, zinc levels in bullheads from our lakes were not high, approximately 50 mg/kg in bone tissue. The mean concentrations of zinc in lacustrine bullheads from the Carnic Alps were 25 mg/kg in muscles and 86 mg/kg in liver what is comparable with our data. Liver is probably the main receptor of zinc in summer, when the amount of ingested metals from diet originates mainly from larvae of midges (Pastorino et al. 2020c). Chironomidae have high concentrations of zinc probably because of their inefficient efflux rates or detoxification mechanisms, as reported in some other studies on aquatic invertebrates (Yu and Wang 2002). Compared to other fish species, bullheads seem to prefer water with relatively higher zinc content (Andreji and Dvorak 2018). Due to floods, zinc is washed out from alpine lakes and is transported to lower transition zones. The trend of consistently declining zinc concentrations in stream populations of bullheads over a 4-year period indicates an absence of biogenic zinc in fish. The average concentration in the bullhead population in the stream has dropped by 70%. Large floods mobilize minerals and sediments in lakes and significantly affect biotic components of stream ecosystems. We noticed that abundance of bullheads in Javorinka significantly decreased (Janiga et al. 2021). Zn is an essential element due to its vital structural catalytic importance in many proteins that play important roles in piscine growth, reproduction, development, vision, and immune function (Bury et al. 2003; Canli and Atli 2003; Malekpouri et al. 2011). Zinc also protects fish from toxic heavy metals (Malik et al. 2010). In the Tatra mountains, a steep rise in air temperature, which is evident from 1980 onwards, leads to unusual heavy storms in summer and consequent large floods. Warming has evidently serious effect on the fish communities including zinc metabolism.

### Rubidium and molybdenum

Rubidium is a lithophile element, and its concentrations in environmental media should mainly reflect natural inputs. High rubidium content occurs in granite soils (Anke et al. 2005). The element is essential to many animals (Anke and Angelow 1995; Anke et al. 2005). Rubidium may accumulate in fish due to its chemical similarity to potassium (Patterson and Settle 1987; Campbell et al. 2005). Acute toxicities of rubidium in vertebrates were observed at higher concentrations, resulting from the replacement of potassium by rubidium (Clayton and Clayton 1994). The concentrations of rubidium and molybdenum in alpine bullheads point to two main phenomena of their cycling in the mountains. First, the metals remain in alpine lakes longer than macrominerals (sulfur, potassium—Table 2); therefore, their ratio to macrominerals is higher in lacustrine fish, and rubidium can compete with potassium in bullhead tissues. Increased uptake and biomagnification of rubidium by fish may result when ambient potassium is low (Peters et al. 1999), suggesting that rubidium concentrations in lacustrine bullheads are dependent not only upon the amount of ambient rubidium but also upon the amounts of other alkali chemical elements in alpine environment. Second, fish absorb and dispose of both metals in the same way; in large floods, bullheads stabilize their original body levels within a year. Annual seasonal flooding is beneficial to river systems and can affect biotic composition, nutrient transport, and sediment distribution, but unpredictable flooding can be disruptive to aquatic organisms and can cause contamination of both aquatic and soil environments (Junk et al. 1989). In fish, rubidium and molybdenum may inhibit spermatogenesis and cause necrosis of spermatogonia (Yamaguchi et al. 2007). Higher concentrations of rubidium are usually found in muscles than in liver, bones, or skin (Espinoza-Quiñones et al. 2011; Plessl et al. 2017; Alti et al. 2022). Molybdenum accumulates mainly in the bones, stomach, kidneys, and liver (Ward 1973), and its elevated levels can be extremely toxic to developing juvenile fish (Davies et al. 2005). Young fish typically have higher levels of molybdenum than adults (Tong et al. 1974; Perinajová et al. 2020; Janiga Jr and Janiga 2023).

### Strontium and calcium

Like rubidium, strontium is also a component of geochemical processes in high mountains. If the rubidium/strontium ratio becomes lower, the indication is that chemical weathering is strong in terrestrial or water ecosystems (Jin et al. 2015). Chemical weathering is very intense in warm and humid climates (Jiang et al. 2018); under such conditions, the strontium content of the water increases rapidly. Gills are probably the major site of strontium uptake, and evidence suggests strontium enters the body using calcium transport systems and may be cleared from the blood very rapidly (Chowdhury and Blust 2011). The metal competes with calcium binding at ion-exchange sites of the cell wall and accumulates mainly in the bones, fins, skin, and less in the gills (Suzuki et al. 1972; Walther and Thorrold 2006; Shotyk et al. 2019). Uptake of strontium ions into fish strongly depends on the concentration of calcium ions in the water; especially, fish bones tend to decrease strontium content in the bones when the concentration of calcium in the water increases (Chowdhury and Blust 2011). The mutual cycling of strontium and calcium in the fish of alpine lakes and streams can be characterized by three important phenomena. The first is related to the leaching of macrominerals of potassium, sulfur, or chlorine from lakes into streams; the ratio of rubidium, strontium, calcium, and molybdenum to macrominerals is therefore higher in lakes (Fig. 6). The second is the similar accumulation of strontium and calcium in fish bodies, as well as their depletion (Fig. 4). This is independent of habitat (Table 2, PC4). The third phenomenon is lower calcium content in lakes and higher in streams, causing the strontium to calcium ratio to be significantly higher in lakes. Our results indicate that strontium in bullhead skin and skull were derived primarily from the ambient water, and water chemistry is the dominant factor controlling the uptake of this metal. Strontium in fish can vary significantly both within and between years (Templeton and Brown 1964), and its amount in the bone may be related to age, with younger individuals showing higher levels than older ones (Speckman and Norris 1964).

The release of toxic metals by human activities to the atmosphere and consequently to the soil and freshwater of alpine ecosystems is a global problem that threatens wild fauna including alpine bullheads. Our results insist on the importance of understanding toxic metal flux variability in the mountains with regard to weathering response. The present study demonstrated that metal loading to alpine lakes has probably a significant effect on fish populations in mountain streams, even for a species that eliminates the effects of flooding and other environmental changes quickly.

## Data Availability

The datasets used and analyzed during the current study are available from the corresponding author on reasonable request.
